# GnRH-Based Immunocastration Vaccines: Comparative Analysis and Role in Boar Taint Reduction

**DOI:** 10.3390/vaccines14070641

**Published:** 2026-07-21

**Authors:** Jisang Kim, Mariusz Skwarczynski, Rachel J. Stephenson

**Affiliations:** School of Chemistry and Molecular Biosciences, The University of Queensland, St. Lucia, QLD 4072, Australia; jisang.kim@student.uq.edu.au (J.K.); m.skwarczynski@uq.edu.au (M.S.)

**Keywords:** immunocastration, boar taint, GnRH vaccine, androstenone and skatole, Improvac, GonaCon

## Abstract

Boar taint is a persistent challenge in pork production, caused primarily by the accumulation of androstenone and skatole in adipose tissue. It affects 5–40% of uncastrated male pigs and is perceived negatively by a substantial proportion of consumers, with skatole detectable by almost all individuals. Traditional surgical castration effectively prevents boar taint but is increasingly criticized on animal welfare grounds. Immunocastration using gonadotropin-releasing hormone (GnRH) vaccines has emerged as a promising alternative, providing reversible suppression of reproductive function while maintaining growth efficiency. This review integrates current knowledge on the biochemical basis of boar taint, evaluates the performance of commercial vaccines such as Improvac^®^ and GonaCon^®^, and examines economic, regulatory, and consumer factors influencing adoption. Immunocastration presents limitations due to variability in immune responses, the need for multiple doses, and challenges related to public perception. To address these challenges, intensive research is underway on single-dose formulations, precision adjuvants, and advanced delivery technologies, all of which are expected to drive the next generation of immunocastration vaccines.

## 1. Introduction

Boar taint is an undesirable odour and flavour in pork derived from entire (uncastrated) male pigs and represents one of the most significant barriers to the widespread use of intact males in pig production [1]. Boar taint arises from the accumulation of lipophilic compounds in adipose tissue, primarily androstenone, a steroidal pheromone, and skatole, a microbial by-product of tryptophan degradation with a faecal odour [2]. When pork meat is heated during cooking, these compounds volatilize, producing urine-, faecal-, or sweat-like aromas that negatively impact consumer acceptance [3,4].

The economic importance of boar taint is considerable and it is typically present in the meat of 5–40% of uncastrated male pigs [5]; however, this problem is nonexistent or minor in meat from castrated pigs [4]. Consumer perception of boar taint varies considerably between compounds. Sensitivity to androstenone is highly heterogeneous; around 40–50% of the human population are partially or completely anosmic to it, while the remainder perceive it often as unpleasant, though occasionally as neutral or even attractive. Women tend to show higher sensitivity than men, with studies reporting 18–31% of consumers being highly sensitive [6,7,8]. In contrast, skatole is detected by approximately 99% of consumers at very low concentrations and is consistently perceived as unpleasant. These two compounds interact synergistically, with skatole often dominating overall odour perception unless individuals are highly sensitive to androstenone, in which case androstenone becomes the key determinant of rejection [3]. Consequently, consumer acceptability depends on both compound concentrations and individual sensory sensitivity. Notably, reported sensory thresholds vary, with androstenone typically detectable at ~500–1000 ng/g fat and skatole at ~100–200 ng/g fat [9,10,11,12]. However, these thresholds are influenced by factors such as genetic variability, cultural differences, and cooking methods. Despite extensive research, variability in consumer responses and inconsistencies in sensory methodologies continue to hinder standardisation and reliable risk assessment [1]. Hence, further research is needed to clarify how cooking conditions influence the perception of boar taint. Additionally, consumer studies are constrained by limited awareness and familiarity, as pork from sexually mature boars is rarely encountered in the market, leaving most consumers without prior exposure to its characteristic odour.

Traditionally, surgical castration, performed within the first week of life and involving the removal of the testes, has been used to mitigate boar taint [1], reduce aggressive behaviour, and promote fat deposition desirable for certain pork products. However, this practice is now widely recognized as a major animal welfare concern due to the acute pain and stress it induces, as well as potential long-term physiological consequences [1,13]. These impacts are evidenced by pronounced behavioural and physiological responses during the procedure (e.g., screaming, resistance, elevated stress hormones, elevated heart rate) [14]. Furthermore, surgical castration is associated with additional risks, including infection and increased pre-weaning mortality. Although anaesthesia effectively reduces intraoperative pain and analgesia alleviates postoperative discomfort, neither approach alone provides comprehensive welfare protection. Their combined use, while more effective, increases both costs and labour. Despite these concerns, castration remains prevalent in major pork-producing regions, driven largely by meat quality requirements and consumer preferences, as pork from intact males is more prone to boar taint and certain quality defects. For example, it is estimated that approximately 75–93% of commercial male piglets are still surgically castrated in countries such as Germany, France, and Denmark [15]. The practice has also persisted due to outdated assumptions that neonatal animals experience reduced pain. Alternatives to conventional castration include the use of anaesthesia and analgesia during the procedure or the avoidance of surgical castration altogether. While pain mitigation strategies improve animal welfare without compromising meat quality, they introduce additional costs and do not eliminate all associated risks. Consequently, legal requirements for pain mitigation during surgical piglet castration vary considerably between countries, with differences in the mandatory use of anaesthesia, analgesia, or both, as well as the age at which these requirements apply.

Avoiding castration may improve animal welfare by eliminating surgical pain; however, it can also lead to management challenges, including increased aggression and harmful social behaviours among intact males. Immunocastration has emerged as a promising alternative, though its adoption is limited by variability in response, the need for repeated dosing, and ongoing concerns around consumer acceptance. Growing societal awareness of animal welfare has driven legislative and policy developments at both regional and global levels, including the recognition of animal sentience and efforts to strengthen welfare standards. Within the European Union (EU), stakeholders committed to phasing out surgical castration by 2018; however, implementation has been uneven and no harmonized alternative has been widely adopted [1]. Divergent national approaches and market considerations have impeded progress despite ongoing policy support.

## 2. Biochemical and Physiological Basis of Boar Taint

### 2.1. Major Compounds and Sensory Thresholds

**Figure 1:**
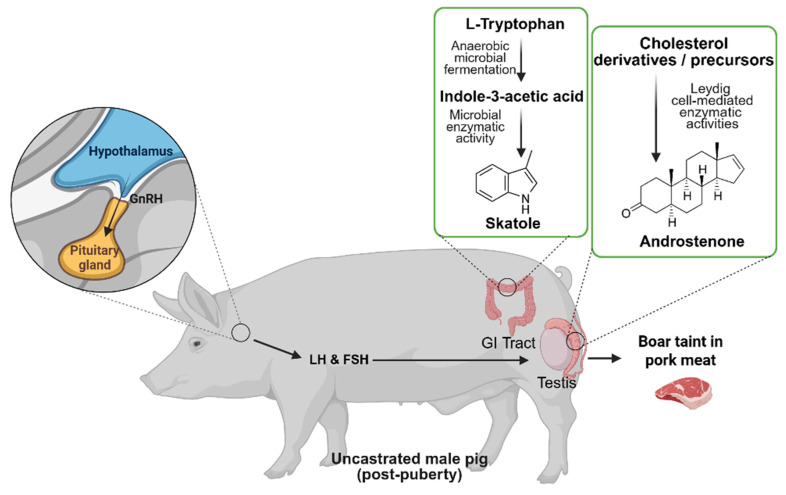
Schematic representation of the biological pathways contributing to boar taint in uncastrated post-pubertal male pigs. Androstenone is synthesised in the testes from cholesterol precursors under hypothalamic–pituitary–gonadal regulation (luteinising hormone [LH], follicle-stimulating hormone [FSH], and gonadotropin-releasing hormone [GnRH]), while skatole is produced in the gastrointestinal tract through microbial degradation of tryptophan. Both compounds accumulate in adipose tissue and contribute to undesirable odours in pork when heated.

Boar taint results from the accumulation of two primary compounds in entire male pigs, androstenone and skatole, with other substances such as indole contributing to a lesser extent. Each of these compounds originates from distinct physiological and microbial pathways and differs markedly in their metabolism, sensory properties, and contribution to overall taint perception (Figure 1). Due to the impact on pork quality, these compounds were subject to regulatory attention in the EU (Regulation 852/2004) where meat exhibiting a pronounced ‘sexual’ odour was deemed unfit for human consumption, although this provision was removed when the regulation was repealed and replaced by Regulation (EU) 2017/625, which no longer explicitly references sexual odour [1].

#### 2.1.1. Androstenone

Androstenone (5α-androst-16-ene-3-one; Figure 1) is a testicular steroid produced in the Leydig cells via pathways shared with testosterone biosynthesis and regulated by the hypothalamic–pituitary–gonadal (HPG) axis [1]. Physiologically, its primary role is to signal reproductive maturity in intact males (boars) and elicit mating behaviour and endocrine responses in females (sows). Due to its lipophilic nature, androstenone is transported in the bloodstream to peripheral tissues, where it preferentially accumulates in adipose tissue. Its metabolism is relatively slow, largely due to the limited activity of key enzymes (e.g., 3β- and 3α-hydroxysteroid dehydrogenases) in hepatic and extrahepatic tissues. This inefficient clearance promotes its persistence in fat, contributing to the characteristic urine- or sweat-like odour associated with boar taint.

#### 2.1.2. Skatole

Skatole (3-methylindole; Figure 1), in contrast, is produced in the hindgut through microbial degradation of l-tryptophan, via intermediates such as indole-3-acetic acid [1,2]. Its formation is influenced by dietary tryptophan availability, gut microbiota composition, and intestinal conditions, including digestive transit time and epithelial cell turnover, which increase substrate availability for bacterial metabolism. Following absorption into the bloodstream, skatole is transported to the liver where it is metabolized by cytochrome P450 enzymes (known to play a predominant role in drug and xenobiotic metabolism) into water-soluble products for excretion [2,16]. However, excessive microbial production or impaired hepatic detoxification leads to accumulation in adipose tissue, where it produces a strong faecal odour.

Notably, skatole metabolism is closely linked to endocrine status. Testicular steroids suppress hepatic enzymes responsible for skatole clearance, resulting in higher skatole accumulation in intact males compared to castrates or females. This interaction highlights the interplay between microbial production and host physiology. Accordingly, interventions that reduce testicular activity, such as immunocastration, not only decrease androstenone synthesis but also enhance hepatic skatole metabolism. Additionally, variability in skatole metabolism has led to the identification of metabolic biomarkers associated with efficient clearance or increased accumulation, further underscoring the complexity of its regulation [17].

#### 2.1.3. Indole

Indole (2,3-benzopyrrole), another microbial metabolite of tryptophan, contributes minimally to boar taint compared to androstenone and skatole [5]. Although present at lower concentrations, indole has been shown to correlate with sensory scores and may contribute additively to overall odour perception. Nevertheless, its impact is considered secondary, and most control strategies primarily target androstenone and skatole.

Collectively, the dual origin of boar taint, from endocrine (androstenone) and microbial/endocrine (skatole and indole) sources, highlights its multifactorial nature and explains why single-pathway interventions (for example regulation of skatole level by dietary changes [2]) often show inconsistent outcomes. Effective control strategies must therefore simultaneously target both testicular steroidogenesis and microbial metabolite accumulation. Among the available approaches, immunocastration is uniquely positioned to address both pathways, providing a mechanism-based solution to boar taint suppression. The following section examines the biological basis and functional consequences of GnRH-targeting immunocastration.

### 2.2. Modulators of Boar Taint

Boar taint is a multifactorial and highly variable trait influenced by biochemical, genetic, and environmental factors, which together contribute to its inconsistent occurrence in commercial production systems.

#### 2.2.1. Genetic

The genetic background of pigs plays a major role with clear breed differences in susceptibility (Table 1) [18]. For example, Duroc pigs typically exhibit higher androstenone concentrations, while breeds such as Meishan show comparatively low levels. Other breeds, including Large White, Landrace, Pietrain, and Hampshire, display moderate and variable susceptibility, with considerable within-breed variation driven by selection, nutrition, and management.

Here, genetic variation strongly influences the production, metabolism, and accumulation of boar taint compounds, as well as the degree of sexual maturity at slaughter. Both androstenone and skatole show moderate to high heritability, making genetic selection a promising long-term, non-invasive, and welfare-friendly strategy for reducing boar taint while supporting the shift toward raising entire males. Selective breeding programs, including genomic selection and the use of genetic markers, have already produced low-taint pig lines (e.g., by Topigs Norsvin [Norway] and Nucleus [France]) [13]. Recent research has also identified specific genomic regions and candidate genes (e.g., *CDK4*, *CRHBP*, *CTDSP2*, *CYP27B1*, and *SDR4E1*) associated with boar taint [22]. Breed-related differences have also been shown to significantly influence androstenone accumulation in pigs, where genetic line affects both baseline levels and responses to production factors [1,23]. For example, entire males from Pietrain × Duroc × Large White crosses exhibit substantially higher androstenone concentrations in backfat compared to Pietrain × Large White or pure Pietrain lines, while immunocastrated pigs show no such breed-specific variation (Table 1).

This variability in dietary, husbandry, environmental, and genetic factors highlights the complex interactions between endocrine regulation, microbial metabolism, and environmental influences in determining boar taint. While individual strategies may reduce specific compound concentrations, particularly skatole, they rarely address both major contributors simultaneously. This limitation underscores the need for integrated approaches targeting the underlying hormonal regulation of boar taint, with immunocastration emerging as a promising strategy capable of modulating both endocrine and metabolic pathways.

#### 2.2.2. Husbandry

Husbandry practices have been shown to strongly influence boar taint levels [24]. For example, social stress in group-housed entire males has been shown to increase aggression and mounting behaviour, associated with elevated androstenone [1,25]. Here, aggression and dominance are linked to androgen activity and may promote earlier puberty, though direct relationships between behaviour and taint remain unclear [13]. Further, housing conditions are particularly important for skatole, where poor hygiene, high stocking density, and contact with faeces can increase its absorption and deposition in fat, while clean environments help reduce its levels [26]. Pre-slaughter handling and transport can further elevate boar taint compounds, as well as seasonal and physiological factors. Pigs raised in autumn/winter were shown to reach puberty earlier due to shorter daylight exposure, leading to higher hormone levels and increased skatole, although effects on androstenone are less consistent [13,24]. Slaughter weight and age also influence boar taint, as heavier pigs are generally more sexually mature; however, correlations between carcass weight and androstenone or skatole levels are typically weak within common commercial ranges [13,27]. Overall, housing, management, and handling conditions are key environmental factors contributing to variability in boar taint.

#### 2.2.3. Dietary

Environmental and management factors further modulate boar taint development. Among these, dietary composition is a key determinant, particularly of skatole synthesis in the hindgut. Diets enriched with fermentable carbohydrates, such as inulin (from chicory), sugar beet pulp, and raw potato starch, have been shown to reduce skatole production by altering gut microbiota activity, limiting tryptophan availability, and decreasing intestinal cell turnover [28,29,30]. Several studies have demonstrated reductions in skatole levels in the intestine and adipose tissue with these dietary interventions, although results remain inconsistent depending on inclusion levels, feeding duration, and experimental conditions. Other approaches, including manipulation of dietary protein levels or the use of adsorbents such as activated carbon, have shown limited or no consistent effects [31]. Hydrolysable tannins may reduce skatole concentrations modestly, but their impact on sensory perception is often minimal, and higher doses may negatively affect food intake. The use of antibiotics to suppress skatole-producing bacteria has proven effective experimentally but is no longer permitted in the European Union [32,33]. In contrast, nutritional strategies to reduce androstenone are limited, as its production is primarily regulated by endocrine mechanisms. One practical approach is to promote rapid growth, allowing pigs to reach slaughter weight before sexual maturity and thereby limiting androstenone accumulation. This strategy is widely used in systems producing lighter carcasses [13].

## 3. Boar Taint Control by Immunocastration

**Figure 2:**
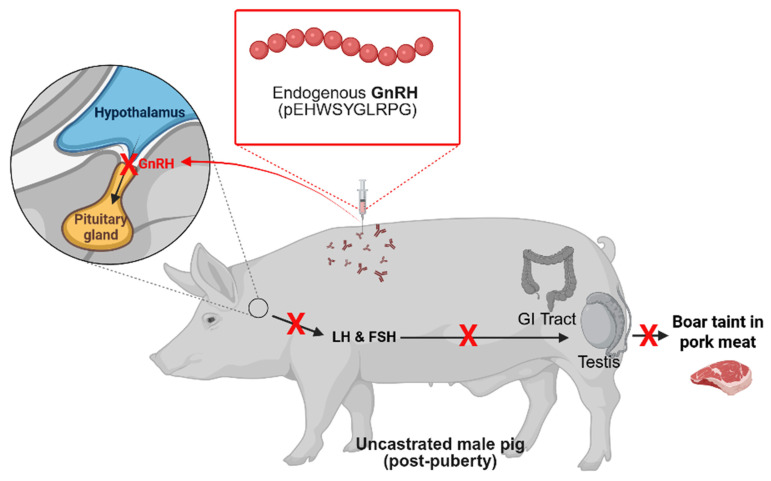
Schematic illustration of immunocastration in post-pubertal male pigs. Vaccination induces antibodies against endogenous gonadotropin-releasing hormone (GnRH), disrupting hypothalamic–pituitary–gonadal regulation signalling and suppressing luteinising hormone (LH) and follicle-stimulating hormone (FSH) release. This inhibition reduces testicular function and downstream androstenone production, as well as indirectly lowering skatole accumulation, thereby preventing boar taint in pork.

Immunocastration is a welfare-friendly alternative to surgical castration that controls boar taint through active immunization against gonadotropin-releasing hormone (GnRH) (formerly known as GnRF or LHRH), the central regulator of the HPG axis. GnRH, a decapeptide (pGlu-His-Trp-Ser-Tyr-Gly-Leu-Arg-Pro-Gly-NH_2_) produced by the hypothalamus, stimulates the secretion of luteinizing hormone (LH) and follicle-stimulating hormone (FSH), which regulate testicular steroidogenesis and spermatogenesis (Figure 2). Interestingly, due to their reversible contraception effects, GnRH vaccines have also been investigated for human applications as a therapeutic vaccine against prostate cancer [34,35].

Due to the small size and low immunogenicity of GnRH as a self-peptide, effective vaccines require conjugation to an immunogenic carrier protein and formulation with an adjuvant to elicit a robust immune response. Following vaccination, anti-GnRH antibodies bind endogenous GnRH, reducing its bioavailability below the threshold required to sustain activation of the HPG axis (Figure 2). Consequently, LH and FSH secretion is suppressed, leading to inhibition of Leydig cell function, reduced testosterone production, and disruption of spermatogenesis. This endocrine suppression becomes pronounced after the booster vaccination, when antibody titres peak, resulting in a functional shutdown of the HPG axis and testicular activity. As a result, production of testicular steroids declines rapidly. Because androstenone is synthesized in Leydig cells and accumulates in adipose tissue, its concentrations typically fall below sensory or even analytical detection thresholds within weeks of effective immunization.

Immunocastration also indirectly reduces skatole accumulation. Although skatole is produced in the hindgut via microbial degradation of tryptophan, its systemic levels are regulated by hepatic metabolism. Testicular steroids normally inhibit cytochrome P450 enzymes responsible for skatole clearance; thus, suppression of androgen production enhances hepatic metabolism and reduces skatole deposition in fat. Consequently, skatole levels in immunocastrated animals often decline to those comparable with surgically castrated pigs.

In addition to biochemical changes, immunocastration induces behavioural and production-related effects consistent with reduced androgen activity. Following the second vaccination, aggression, mounting, and sexual behaviours decrease, improving animal welfare and reducing injury incidence. Growth performance also shifts from animals that initially retain the efficiency and lean growth of entire males to following endocrine suppression, and metabolism transitions toward that of castrated pigs, with increased feed intake and altered fat deposition.

Several GnRH immunocastration vaccines exist across species, including Improvac^®^/Improvest^®^, Bopriva^®^, Equity^®^, and GonaCon^®^ (Table 2) [36]. These vaccines have been extensively tested for dosage, immunization intervals, and potential reversibility in a large number of domestic animal species, such as male pigs, mares and stallions, male goats, feral female cattle, male cats or male dogs, as well as exotic companion and wildlife animals (e.g., elephants, elk, lions, giraffes) [37,38,39]. However, in Europe only Improvac^®^ is approved for pigs. Despite this, its adoption in the EU is low (about 2.8% of male pigs), largely due to uncertainty and limited experience among stakeholders [36,40,41,42,43]. In contrast, the vaccine usage is more prevalent in Belgium (~15%), while in Brazil and Australia it exceeds 50%.

### 3.1. Improvac

The first anti-GnRH product approved for widespread use in the swine industry was Improvac^®^. Improvac is marketed by Zoetis, Inc. and is known to have been commercially available in over 60 countries [51]. First approved in Australia in 1988 and later in Europe in 2009, it is marketed globally under various trade names (e.g., Improvest^®^, Innosure^®^, Vivax^®^). Notably, Improvac^®^ is the sole product licensed for swine immunocastration in Europe to reduce the boar taint in pork [52].

Improvac^®^ consists of a synthetic GnRH analogue conjugated to diphtheria toxoid (DT) as a carrier protein and formulated with diethylaminoethyl (DEAE)-dextran adjuvant to enhance immune activation. Improvac^®^ follows a two-dose regimen, with the first dose priming the immune system and the second dose, administered approximately 4–6 weeks later, inducing high-titre anti-GnRH antibodies. This results in rapid suppression of LH/FSH secretion, reduced testosterone production, and consequent inhibition of androstenone synthesis and indirect reduction of skatole accumulation. Consequently, effective suppression of testosterone and testicular function within 1–2 weeks post the second immunization, androstenone concentrations fall rapidly, typically reaching below sensory detection thresholds prior to slaughter, and the effect is reversible, with reproductive function gradually returning after antibody titres decline [53,54,55]. The duration of effective suppression is limited (typically ~18–22 weeks), reflecting the waning of antibody titres, necessitating precise timing of the second vaccination relative to slaughter [54,56,57]. Studies evaluating vaccination timing show that administering the second dose ~3–6 weeks before slaughter optimizes boar taint suppression while maintaining favourable carcass traits [58]. Importantly, research demonstrates that delayed second vaccination prolongs the entire male growth phase, improving feed efficiency, and earlier second vaccination increases fat deposition but ensures maximal taint control [58,59,60]. This tuneable response is a major advantage of immunocastration compared with surgical castration, allowing producers to strategically balance growth performance and meat quality.

A key limitation of Improvac^®^ is the transient nature of the immune response, necessitating booster vaccination to achieve effective and sustained endocrine suppression. Early studies demonstrated that a single immunization produces insufficient antibody titres, whereas a booster dose reliably induces castration-like physiological changes, including reduced testosterone secretion and regression of reproductive tissues [55]. These findings underpin the standard two-dose vaccination protocol, consisting of an initial priming injection followed by a second activating dose administered 4–6 weeks prior to slaughter. Further, Improvac^®^ vaccination effectiveness varies between farms and individual animals, with up to 3% of boars showing little or no response [52]. Factors such as stress, poor welfare, illness, or vaccination errors can weaken the immune response. Animals that do not show reduced scrotal size may be non-responders, and a third vaccine dose may be considered.

### 3.2. GonaCon^®^

GonaCon^®^ was developed by the U.S. Department of Agriculture’s Wildlife Services National Wildlife Research Center and is primarily used for fertility control in wildlife populations (Table 2) [61]. It consists of a GnRH analogue conjugated to keyhole limpet hemocyanin (KLH) and formulated with AdjuVac™, a highly potent water-in-oil emulsion containing inactivated *Mycobacterium avium* [62,63]. The strong immunostimulatory properties of AdjuVac™ enable single-dose vaccination, producing long-lasting immune responses and, in many species, multi-year suppression of reproductive function. This makes GonaCon^®^ particularly suitable for free-ranging species where repeated handling is impractical [64]. However, despite its efficacy, GonaCon^®^ has limited applicability in commercial livestock production due to high incidence of injection-site reactions and granuloma formation and presence of mycobacterial components that may interfere with tuberculosis diagnostics. Consequently, while GonaCon^®^ demonstrates the feasibility of long-lasting single-dose immunocastration, its use remains largely restricted to wildlife management systems, whereas Improvac^®^ remains the dominant vaccine in pig production (Table 2).

### 3.3. Equity^®^

Equity (Zoetis) Equity^®^ is a protein-based anti-GnRH vaccine for horses, mainly used in Australia to suppress reproductive hormones, preventing oestrus in mares and reducing stallion behaviour (Table 2) [37,65]. Administered in two doses four weeks apart, it effectively reduces hormone levels and oestrus-related behaviour for at least three months. Common side effects include injection-site reactions such as swelling and pain. Although widely used in Australia and New Zealand for oestrus control in wild horses, it was not licensed in the United States and has not been commercially available since 2010 [66,67].

### 3.4. Bopriva^®^

Bopriva^®^ (Zoetis), similar to Improvac^®^ but designed for use in cattle, was introduced in 2007 in Australia and New Zealand [68,69], and is currently the only such product available for bulls [64]. Bopriva, like Improvac^®^, is composed of a synthetic GnRH analog coupled with the DT to act as an immunogenic carrier protein. Bopriva also makes use of the adjuvant DEAE dextran, but unlike Improvac, Bopriva^®^ couples DEAE dextran with another adjuvant system, AdvaSure^®^. Bopriva^®^ requires two doses: the first primes the immune system, while the second triggers a strong antibody response within 7–14 days. Tested side-by-side in boars, Improvac^®^ and Bopriva^®^ both increased GnRH antibody levels after the second dose, leading to a sharp drop in testosterone and reduced sperm production [64]. However, Bopriva^®^ had a stronger and longer-lasting effect where testosterone remained suppressed for a period of 10 weeks and sperm production and testicular development were more severely impaired. In contrast, Improvac^®^-treated boars began to recover by around 8 weeks. Overall, while both vaccines reduced testicular function, Bopriva^®^ produced a more prolonged and complete immunocastration profile. The contrasting profiles of Improvac^®^ and GonaCon^®^ highlight a fundamental trade-off in GnRH vaccine design (Table 2), where achieving high immunogenicity and long duration of action must be balanced against safety, tissue reactivity, and regulatory acceptability. Current commercial success favours Improvac^®^, which provides reliable efficacy with minimal carcass defects, despite the requirement for booster dosing. In contrast, GonaCon^®^ demonstrates superior durability but is constrained by safety and regulatory limitations in food-producing animals.

Despite strong efficacy, implementation of immunocastration in commercial systems presents practical challenges, including the requirement for two injections, increasing labour and handling, the need for precise timing relative to slaughter, and variability in immune response between animals. These factors directly influence consistency and adoption at scale, particularly in large production systems where logistical simplicity is critical.

### 3.5. Influence of Immunocastration Timing

Over recent years, the average weight of pigs at slaughter in Australia has increased (Pig Stats, 1998), driven by the efficiencies associated with the slaughter of heavier pigs [56]. The timing of the second Improvac^®^ vaccination is a critical determinant of both boar taint suppression and carcass composition. Normal vaccination procedure involves two injections at least 4 weeks apart; the first injection is given after 8 weeks of age and the second injection 4–6 weeks before slaughter, although boar taint control is still effective as much as 10 weeks after the second injection according to the manufacturer [70]. Administering the second dose 2–4 weeks before slaughter ensures maximal suppression of androstenone and skatole. Earlier administration leads to increased feed intake, greater fat deposition, and reduced lean efficiency, whereas later administration preserves lean growth but risks incomplete taint suppression if antibody titres have not fully developed [71,72].

Werner et al. found that early immunization of pigs (at weeks 3 and 7) with Improvac^®^ was feasible and easily integrated into standard management practices without negatively affecting growth performance, behaviour, or welfare [71]. At the end of the two-dose study, all animals were effectively rendered infertile; however, compared to the standard vaccination schedule, early immunized pigs had higher and more variable testosterone, androstenone, and skatole levels at slaughter, indicating less consistent control of boar taint. Meat quality and fatty acid composition were not significantly affected by the immunization timing.

Further, Brunius et al. compared early (10 and 14 weeks) and standard (16/20 weeks) Improvac^®^ vaccination schedules, with all pigs slaughtered at 25 weeks, alongside surgically castrated and entire males [70]. In both vaccination groups, antibody titres increased rapidly after the second dose, leading to marked reductions in testosterone and skatole to levels similar to surgically castrated pigs. At slaughter, vaccinated pigs had low androstenone, skatole, and oestradiol levels, while entire male pigs presented elevated levels of these compounds. Reproductive organs were reduced in size in vaccinated pigs, particularly in the early vaccination group. Overall, early vaccination (10/14 weeks; prime/boost) was as effective as the standard schedule (16/20 weeks; prime/boost) in suppressing boar taint and testicular function.

Dunshea et al. evaluated the effectiveness of Improvac^®^ in controlling boar taint in pigs slaughtered at 23 or 26 weeks [56]. Vaccination, given 8 and 4 weeks before slaughter, successfully induced strong anti-GnRH antibody responses, leading to reduced testicular size, low testosterone levels, and suppression of reproductive function. Improvac^®^ completely eliminated boar taint, with all treated pigs showing androstenone and skatole levels below detection or threshold limits, comparable to surgically castrated pigs, while many untreated boars showed high levels. In addition, immunocastrated pigs grew faster after the second vaccination, likely due to reduced aggressive and sexual behaviour, and showed better feed efficiency and leaner carcasses than barrows (pigs castrated before reaching breeding age). The vaccine was well tolerated, with no observed injection-site issues. A comparative summary of the principal findings from these studies is presented in Table 3 below.

Collectively, the studies summarised in Table 3 demonstrate that Improvac^®^ effectively induces immunocastration, resulting in rapid suppression of testosterone concentrations and successful control of boar taint following the second vaccination. Despite differences in study design, genetic background, and vaccination schedules, all studies reported reduced reproductive function and androstenone and skatole concentrations that remained below sensory thresholds in almost all vaccinated pigs. Both early and standard vaccination schedules elicited strong anti-GnRH antibody responses and maintained low testosterone concentrations until slaughter, although the early schedule showed slightly lower antibody titres and greater variability in endocrine and boar taint outcomes. In contrast, the standard manufacturer-recommended schedule generally produced more consistent testosterone suppression and less variation in androstenone and skatole concentrations at slaughter. Evaluation across three genetically distinct sire lines further confirmed the efficacy of Improvac^®^, although a small proportion of carcasses exceeded established androstenone or skatole cut-off values. Nevertheless, no carcasses were identified as boar tainted by sensory assessment in that study. Overall, these findings highlight vaccination timing as an important factor influencing the consistency of treatment outcomes while supporting Improvac^®^ as an effective strategy for controlling reproductive function and boar taint under commercial production conditions [56,70,71,73].

## 4. Global Adoption, Regulation, and Consumer Perception of Immunocastration

### 4.1. Adoption of Entire (Uncastrated) Pigs

Raising entire male pigs eliminates the welfare concerns associated with surgical castration, as it avoids the pain and stress of the procedure [1,13,14,74]. It also offers economic and sustainability advantages, including reduced labour and processing costs, improved feed efficiency, lower carbon footprint, and increased lean meat deposition due to hormonal effects during puberty. These factors generally enhance production efficiency and can improve carcass value. However, this approach presents several challenges. Entire males exhibit increased aggressive and sexual behaviours, leading to injuries, higher stress levels, and more demanding management conditions. Meat quality can also be compromised, with lower intramuscular fat, reduced water-holding capacity, greater toughness, and poorer suitability for processed products, particularly dry-cured items [14]. Additionally, their fat is more unsaturated and softer, which negatively affects handling and product quality. Notably, a key limitation remains the risk of boar taint, which varies depending on genetics, management, and slaughter conditions. While behavioural traits are not reliable predictors of taint compounds, targeted management strategies can help reduce their occurrence. The adoption of entire male production varies widely across Europe. It is common in countries such as the United Kingdom, Ireland, Spain, Portugal, and Greece, where pigs are often slaughtered before puberty at lighter weights. In contrast, uptake is more limited in countries like France, Germany, and Belgium, reflecting ongoing market and production challenges [14,74].

### 4.2. Global Adoption of Immunocastration

Immunocastration has been developed as a welfare-friendly alternative to surgical castration, but its adoption varies considerably across regions (Table 4) [42]. Although the technology has been commercially available for over a decade and demonstrates clear benefits in terms of feed efficiency, growth performance, meat quality, and effective control of boar taint [75], its global uptake remains uneven. High levels of adoption are observed in countries such as Brazil, Canada, and Australia, where production systems and market structures have supported its implementation. In contrast, adoption within Europe has been relatively limited, despite strong policy support, including the European Declaration promoting alternatives to surgical castration. Variability in stakeholder acceptance, market requirements, and supply-chain coordination has constrained wider implementation, and immunocastration is unlikely to become a universal solution across the EU. Similarly, in the United States, although approved products are available (e.g., Improvest^®^, Table 4), uptake remains low due to ongoing concerns regarding market acceptance and processing logistics.

These patterns indicate that the limited adoption of immunocastration is not due to technical or biological shortcomings, but rather to socio-economic and supply-chain factors. Farmers often express caution due to the need for additional training, management adjustments, and clearer economic data on costs and returns. At the same time, consumer awareness of immunocastration remains relatively low and differs across regions. Acceptance is primarily shaped by perceptions of animal welfare, meat quality, and food safety, with some consumers perceiving trade-offs between these attributes that influence willingness to pay. Notably, in some instances, administering immunocastration vaccines is much easier than the procedure of physical castration, and obviously avoids the associated risk of injury and treatment cost [76]. Consequently, effective communication plays a critical role in adoption. Transparent, targeted information strategies, addressing both industry stakeholders and consumers, are essential to improve understanding, reduce misconceptions, and support informed decision-making. Together, these factors highlight that the broader uptake of immunocastration will depend on coordinated efforts across the supply chain rather than further technological development.

**Table 4:** International regulatory status and commercial adoption of Improvac^®^, and national variations in pain-mitigation obligations for surgical piglet castration [77,78,79,80,81,82,83,84].

Country	Regulatory Status	National-Scale Adoption of Improvac^®^	Legal Obligations * for Surgical Piglet Castration; Mandatory Age
Australia	Licensed	High for large producers; Variable for smaller producers	Anesthesia; >21 days
Canada	Licensed	Used in integrated supply chains; Variable for smaller producers	Anesthesia and analgesia; >10 days
China	Registered	Limited	No legal mandates
Europe (EU)	Authorized	Increasing in some countries	Anesthesia and analgesia; >7 days
Japan	Registered	Moderate	Not explicit statement for use
New Zealand	Licensed	Increasing for large producers; Variable for smaller producers	Analgesia; all ages
Republic of Korea	Registered	Limited	Not specified
United States	FDA-approved (Improvest^®^ **)	Limited	Not specified

FDA: Food and Drug Administration (United States). * Legislative requirements for pain mitigation during surgical piglet castration, including anaesthesia and/or analgesia where applicable. ** United States trade name for Improvac^®^.

### 4.3. Regulatory Framework

Regulation of immunocastration is generally well established, particularly in regions with strong veterinary oversight. In the European Union, Improvac^®^, the most widely used immunocastration vaccine, was authorised by the European Medicines Agency in 2009 for use in male pigs [36,85,86,87]. The vaccine is regulated as a veterinary medicinal product and requires prescription-based administration, ensuring controlled and safe use. Importantly, immunocastration is recognised as an immunological intervention rather than a hormonal treatment, meaning it does not pose risks of hormonal residues in meat products. This distinction is critical for regulatory acceptance and consumer safety assurance. In addition, international regulatory bodies and veterinary organisations increasingly support immunocastration as consistent with animal welfare objectives, particularly in comparison to surgical castration performed without anaesthesia. Despite regulatory approval, policy implementation varies. The EU has promoted a transition away from surgical castration, but differences in national policies, supply-chain requirements, and retailer standards have resulted in heterogeneous adoption patterns across member states. Outside Europe, regulatory approval is also widespread, with immunocastration products licensed in more than 60 countries, highlighting global recognition of the technology’s safety and efficacy.

### 4.4. Animal Welfare and Ethical Considerations

Animal welfare has been a major driver behind the development and promotion of immunocastration. Surgical castration, often performed without anaesthesia, is widely recognised as a significant welfare concern due to pain, stress, and risk of complications [86]. Immunocastration eliminates the need for invasive procedures, thereby reducing acute pain and improving overall welfare outcomes. Immunocastration, as a viable welfare-improving alternative, suppresses sexual and aggressive behaviours while avoiding the negative impacts associated with surgery. However, some welfare considerations still exist, particularly during the period before the second vaccination when animals may exhibit behaviours typical of entire males (e.g., aggression and mounting). From an ethical perspective, immunocastration aligns with broader societal trends favouring non-invasive and reversible interventions in animal production systems. It is often regarded as a compromise between maintaining production efficiency and improving animal welfare, especially in systems where raising entire males may present management challenges.

Interestingly, Baumgartner et al. compared behaviour in male pigs that were either surgically castrated or immunocastrated with a GnRH vaccine under commercial conditions [88]. Immunocastrated pigs were generally more active during the fattening period but showed reduced activity (more lying and sitting) after the second vaccination. Overall levels of aggressive behaviour were similar between treatments; however, aggression decreased significantly after the second vaccination in immunocastrated pigs, unlike in surgical castrates. Mounting behaviour was slightly higher in immunocastrated pigs but remained low, and no differences were found in play or social interactions. The results indicate that immunocastration does not increase behavioural problems and provides comparable or improved welfare outcomes compared to surgical castration.

### 4.5. Consumer Perception and Market Acceptance

Consumer perception remains a key constraint to the widespread adoption of immunocastration, despite its demonstrated welfare and production benefits. Concerns primarily relate to food safety, perceived “hormone-like” effects, and distrust of unfamiliar technologies, all of which can negatively influence purchasing decisions and willingness to pay. These perceptions vary among consumer groups and are shaped by both prior knowledge and communication. While providing information that immunocastration is a vaccine-based method without residue risk can improve acceptance, misconceptions, such as confusion with hormonal treatments, still occur [89].

Consumer awareness of pig production practices is generally limited, which further influences attitudes. For example, only around 40% of consumers are aware of piglet castration practices, yet approximately 60% express a preference for immunocastration over surgical castration [89]. Similarly, a large cross-country European study reported that nearly 70% of consumers preferred vaccination and considered it equally effective for controlling boar taint [89,90]. These findings suggest that stakeholder concerns regarding low consumer acceptance may be overstated. However, consumer responses are not uniform. Perceived health risks play an important role in shaping attitudes and purchasing behaviour. In Italian consumers, immunocastration is generally viewed positively, but varying levels of perceived risk influence willingness to pay, with higher acceptance among younger, rural consumers and those more concerned with animal welfare [91]. Key factors driving consumer decisions include animal welfare, meat quality, and food safety, with some individuals perceiving trade-offs between these attributes.

Sensory studies further highlight the importance of meat quality in consumer acceptance. Across multiple European countries, pork from immunocastrated pigs shows significantly higher acceptance than meat from entire males, which is often rejected due to boar taint. In many cases, sensory attributes such as odour, flavour, and overall liking are comparable to those of surgically castrated pigs [92], supporting immunocastration as an effective strategy to maintain product quality. However, sensitivity to boar taint compounds, particularly skatole, remains a key determinant of consumer rejection.

The influence of expectations and information framing is also evident. Font-i-Furnols et al. identified distinct consumer segments, ranging from supportive to opposed, and demonstrated that acceptance can depend on how information about production methods is communicated [93]. Notably, providing more detailed or complex information does not always significantly alter attitudes, suggesting that consumer perceptions may be relatively stable rather than easily shifted through education alone [94].

Finally, supply-chain actors, particularly retailers, play a critical role in shaping market adoption. Even where consumer acceptance is moderate to high, perceived risks of negative consumer reactions can limit industry uptake. As a result, successful implementation depends not only on actual consumer attitudes but also on stakeholder confidence. Clear, transparent communication regarding food safety, pricing, and eating quality will therefore be essential to reduce misconceptions and support broader adoption of immunocastration.

## 5. Immunocastration Outlook and Innovation

Despite the proven efficacy of commercial immunocastration vaccines against boar taint, several scientific and technological advancements are being explored to address key limitations [1,5,95,96]. One of these objectives is the design of single-dose, long-acting formulations capable of inducing sustained reproductive suppression without the need for booster injections [97]. Achieving this goal depends on improving both antigen (namely a GnRH-carrier protein conjugate) persistence and immune programming (namely efficacy of a vaccine adjuvant).

Recent advances in vaccinology highlight that durable immunity is driven by coordinated control of antigen kinetics, lymphatic trafficking, and germinal centre responses, rather than antigen dose alone. This has reinforced interest in controlled-release platforms, where antigen availability is extended over time to mimic natural infection dynamics and promote long-lived plasma cell formation. In this context, depot-forming systems, water-in-oil emulsions (e.g., AdjuVac™) and biodegradable polymer carriers (e.g., EPV-608) are particularly valuable because they maintain antigen presentation while continuously stimulating innate immune pathways. Along the same lines, vaccine efficacy was boosted through modification of GnRH carrier system. Namely, GnRH conjugated to pig T helper epitope was derived from the swine flu virus, and polymethylacrylate, upon self-assembling into nanoparticles, generated high anti-GnRH IgG titres after both single subcutaneous or oral immunization of pigs [98]. The physicochemical properties of these formulations, including oil and polymer composition and stability, play a critical role in determining both immunogenicity and duration of effect. Collectively, these strategies aim to extend the duration of GnRH suppression while reducing reliance on the current two-dose protocols used in vaccines such as Improvac^®^. However, it is important to note that most of these technologies remain at the experimental stage, where validation in pigs is limited, and few studies have demonstrated consistent, long-term reproductive suppression under commercial conditions.

Modern vaccine delivery technologies further support this strategy. Needle-free injection systems, particularly electromagnetic jet injectors, have gained increasing attention in veterinary medicine [99,100,101]. These devices deliver vaccines intradermally by propelling a high-velocity liquid stream through the skin, enabling precise deposition of antigen within dermal immune cell networks. In addition to improving operator safety by eliminating needlestick injuries, these systems reduce carcass defects associated with needle punctures and remove the risk of broken needles in meat products. Studies in swine have shown that needle-free delivery of vaccines against pathogens such as classical swine fever virus (CSFV), porcine reproductive and respiratory syndrome virus (PRRSV), and porcine circovirus type 2 (PCV2) result in enhanced antibody responses, faster seroconversion, and stronger cell-mediated immunity compared to conventional intramuscular injection [73,102,103,104]. These findings suggest that such technologies may also enhance the performance of immunocastration vaccines by improving antigen targeting to cutaneous antigen-presenting cells.

Alternative non-invasive delivery strategies, including oral and intranasal vaccination, offer practical advantages for large-scale livestock systems by enabling mass administration and reducing animal handling [105]. However, these approaches present significant technical challenges. Intranasal delivery primarily stimulates mucosal IgA responses, which are insufficient for effective immunocastration, as suppression of the HPG axis requires strong systemic IgG responses. Oral vaccines face additional barriers, including degradation by gastric acid, digestive enzymes, and the intestinal microbiota, as well as limited absorption of antigen into systemic circulation. Overcoming these challenges requires advanced formulation strategies, such as encapsulation within liposomes, nanoparticles, or polymer matrices, combined with effective mucosal adjuvants [98]. Despite their theoretical advantages, these technologies remain under development and require further optimisation for application to small, unstable peptide antigens such as GnRH.

Nucleic acid-based vaccines represent another promising frontier. DNA and mRNA platforms offer rapid design, scalability, and the potential to induce strong humoral and cellular immune responses [106,107,108,109]. While DNA vaccines have been explored in wildlife fertility control, and mRNA technologies have demonstrated global feasibility in human vaccination, their application to GnRH immunocastration is more complex. The small size and low intrinsic immunogenicity of GnRH mean that constructs encoding the peptide alone typically fail to induce adequate antibody responses. To overcome this limitation, several strategies are being investigated, including antigen multimerization, incorporation into self-assembling nanoparticle systems, fusion to carrier proteins containing T-helper epitopes, and inclusion of molecular adjuvants or immunostimulatory sequences [109]. Although these approaches show promise, their practical application in livestock systems remains constrained by high production costs, cold-chain requirements, formulation stability challenges, and regulatory considerations.

Precision adjuvant design focuses on using defined particulate systems, saponin-based formulations, polymer carriers, and pattern-recognition receptor agonists to achieve targeted activation of the immune system [110,111]. These systems aim to enhance antigen delivery and promote controlled activation of innate and adaptive immune responses while minimising local tissue reactivity. The goal is to generate strong, durable antibody responses with improved safety profiles, particularly compared to traditional mineral-oil or mycobacterial adjuvants, which are often associated with injection-site reactions. Further, for a balanced immune response, Th2-associated pathways are essential for generating high-titre antibody responses necessary for GnRH neutralisation, while a controlled Th1-associated component enhances antigen presentation and immune priming [110,111,112]. Importantly, excessive pro-inflammatory responses must be avoided to minimise tissue damage and improve carcass quality. Recent veterinary studies suggest that precision adjuvants can achieve more predictable immune activation and reduce adverse effects compared with conventional formulations, highlighting their potential to improve both vaccine efficacy and safety.

Advances in vaccine formulation, delivery systems, and adjuvant design provide a strong foundation for the development of next-generation immunocastration technologies. Addressing current limitations, particularly the need for multi-dose regimens, variability in immune response, and risk of injection-site reactions, will be essential for improving consistency and scalability in commercial production. Ultimately, the successful evolution of immunocastration vaccines will depend on achieving a balance between efficacy, safety, cost-effectiveness, and practical implementation. Continued innovation in these areas will be critical to enhancing animal welfare, improving production efficiency, and strengthening consumer confidence, positioning immunocastration as a key component of sustainable pig production systems.

## 6. Conclusions

Boar taint remains a multifactorial challenge in modern pork production, influenced by endocrine, microbial, genetic, nutritional, and management-related factors. Among the available mitigation strategies, immunocastration represents one of the most comprehensive approaches, as suppression of the hypothalamic–pituitary–gonadal axis simultaneously reduces androstenone production and enhances hepatic clearance of skatole while avoiding the animal welfare concerns associated with surgical castration. Commercial GnRH vaccines, such as Im-provac^®^, have demonstrated effective control of boar taint while maintaining favourable production performance and improving animal welfare. However, broader implementation continues to be influenced by practical considerations, including vaccination logistics, variability in individual immune responses, regulatory requirements, economic factors, and consumer acceptance. These challenges highlight that successful adoption depends not only on biological efficacy, but also on coordinated engagement among producers, processors, regulators, retailers, and consumers.

Despite the substantial welfare and production benefits of immunocastration, several practical limitations remain. Treatment success depends on correct vaccine administration and appropriate timing of the second vaccination, while a small proportion of animals may remain non-responders despite vaccination. Although injection-site reactions are generally uncommon with Improvac^®^, local reactions have been reported for some immunocastration vaccines and should continue to be monitored. Commercial implementation also requires additional labour for booster administration, and optimisation of vaccination timing must balance carcass composition with reliable boar taint control. While earlier immunocastration may provide greater management flexibility and maintain favourable production performance under some production systems, it is generally associated with greater variability in endocrine suppression and boar taint control than the standard two-dose regimen. Furthermore, broader adoption continues to be influenced by retailer acceptance, consumer misconceptions regarding vaccine safety, and the need for practical monitoring strategies to identify treatment failures before slaughter. Future research should focus on optimising vaccination schedules and advancing adjuvant technologies to improve treatment consistency, facilitate effective single-dose immunocastration, and minimise the greater variability currently associated with earlier vaccination strategies.

Continued advances in vaccine design are expected to further strengthen the role of immunocastration in swine production. Improvements in antigen design, adjuvant systems, controlled-release formulations, and alternative delivery platforms may enhance vaccine safety, efficacy, and the magnitude and duration of immune responses while reducing reliance on conventional two-dose vaccination schedules. As these technologies mature and stakeholder confidence grows, immunocastration is well positioned to become an increasingly practical and sustainable strategy for balancing animal welfare, meat quality, and production efficiency. Accordingly, immunocastration is likely to play an important role in continued future pork production systems, supporting the transition toward more welfare-conscious and sustainable approaches to boar taint management.

## Figures and Tables

**Table 1:** Variations in boar taint levels amongst common pig breeds [16,18,19,20,21].

Pig Breed	Genetic Susceptibility to Boar Taint *
Duroc	High; consistently elevated androstenone concentrations and greater boar taint risk.
Landrace	Moderate-high; variable, with some lines showing increased skatole and androstenone levels.
Large White (Yorkshire)	Moderate; typically lower androstenone than Duroc but higher than low-taint breeds.
Hampshire	Moderate; variability across genetic lines, with intermediate taint risk.
Pietrain	Low-moderate; typically lower androstenone levels, associated with lean carcass traits.
Meishan	Low; characteristically low androstenone accumulation.

* Values reflect general breed tendencies. Substantial within-breed variation occurs due to genetic selection, as well as interactions with nutrition, housing, management, and slaughter conditions.

**Table 2:** Comparative summary of GnRH immunocastration vaccines [19,44,45,46,47,48,49,50].

Feature	Improvac^®^	GonaCon^®^	Equity^®^	Bopriva^®^
Manufacturer	Zoetis, Inc. (formerly Pfizer Animal Health, Parkville, Victoria, Australia)	U.S. Department of Agriculture’s Wildlife Services National Wildlife Research Center	Zoetis	Zoetis (formerly Pfizer Animal Health, Parkville, Victoria, Australia)
Antigen	GnRF *-diphtheria toxoid	GnRH-keyhole limpet hemocyanin (KLH)	GnRF *-diphtheria toxoid	GnRF *-protein conjugate
Dose Regimen	Two-dose	Single-dose	Two-dose	Two-dose
Adjuvant	Diethylaminomethyl (DEAE) dextran	AdjuVac™ (*Mycobacterium avium* water-in-oil emulsion)	Synthetic adjuvant (not disclosed)	AdvaSure^®^ (double oil emulsion containing DEAE-dextran and an immune-stimulating complex)
Target Species	Domestic pigs	Wild horses and deer, ground squirrels, prairie dogs, wild pigs, elk, kangaroo, feral cattle	Horses	Cattle
Duration of Effect	~22 weeks	1–2 years (species-dependent)	3–6 months (mares); 6–12 months (stallions)	3–4 months
Regulatory Approval	Australia, New Zealand, Europe, Japan, Brazil, Mexico, South Africa	United States (wildlife only)	Australia and New Zealand **	Australia, New Zealand, Latin America (including Mexico, Brazil, Argentina, and Peru)

* GnRF: Gonadotropin-releasing factor (historical term for GnRH). ** No longer manufactured or commercially available.

**Table 3:** Comparative summary of representative studies evaluating Improvac^®^ vaccination schedules in male pigs.

Study Design and Animals	Vaccination Schedule and Slaughter	Anti-GnRH Antibody Response	Testosterone Suppression	Androstenone and Skatole Level Outcomes	Reference
Commercial 2 × 3 factorial study (*n* = 300 Large White × Landrace cross- bred males; *n* = 200 boars, *n* = 100 barrows) Groups: surgically castrated, placebo-treated and Improvac-treated.	Improvac at 15 and 19 weeks (slaughter at 23 weeks) Improvac at 18 and 22 weeks (slaughter at 26 weeks).	Anti-GnRH anti-bodies detected in all vaccinated pigs after the second dose; absent in controls except one transient non-specific response.	92% (46/50) of vaccinated boars had serum testosterone < 2 ng/mL four weeks after the second vaccination. Testis and bulbourethral gland weights decreased by ~50%.	All vaccinated pigs remained below sensory thresholds for androstenone (1.0 μg/g fat) and skatole (0.20 μg/g fat), indicating effective control of boar taint.	[56]
Comparative study of *n* = 192 crossbred male pigs (Swedish Yorkshire dams × Swedish Landrace or Swedish York-shire sires) allocated to surgically castrated, early-vaccinated, standard- vaccinated or entire male groups.	Early vaccination: 10 and 14 weeks of age (11 weeks be-fore slaughter).	Antibody titres increased rapidly after the second vaccination, although titres were slightly lower than the standard schedule (at 2 and 4 weeks after the second vaccination).	Plasma testosterone de-creased rapidly after the second vaccination and remained low until slaughter.	Mean fat androstenone: 169 ng/g (90–318 ng/g); mean fat skatole: 25 ng/g (20–32 ng/g). No vaccinated pigs exceeded sensory thresholds (1 μg/g androsterone; 0.20 μg/g skatole).	[70,71]
Comparative study of *n* = 192 crossbred male pigs (Swedish Yorkshire dams × Swedish Landrace or Swedish York- shire sires) allocated to surgically castrated, early-vaccinated, standard-vaccinated or entire male groups.	Manufacturer-recommended vaccination: 16 and 20 weeks of age (~5 weeks before slaughter).	Rapid increase in antibody titres after the second vaccination; titres slightly higher than the early-vaccination schedule (detailed above).	Plasma testosterone declined rapidly after the second vaccination and remained low until slaughter.	Mean fat androstenone: 169 ng/g (90–317 ng/g); mean fat skatole: 26 ng/g (21–33 ng/g). No vaccinated pigs exceeded sensory thresholds (1 μg/g androstenone; 0.20 μg/g skatole).	[70,71]
Longitudinal study of *n* = 55 male pigs from Belgian Piétrain, French Piétrain, and Cana dian Duroc sire lines.	First vaccination at ~50 kg (8 weeks be-fore slaughter); second vaccination at ~80 kg (≥27 days before slaughter).	Not directly assessed.	Serum testosterone de-creased significantly after the second vaccination across all sire lines.	One carcass (1.9%) exceeded the androstenone cut-off (2000 μg/kg) and two carcasses (3.7%) exceeded the skatole cut-off (250 μg/kg); no carcasses were classified as boar tainted by sensory assessment.	[73]

## Data Availability

No new data were created or analyzed in this study. The original contributions presented in this study are included in the article. Further inquiries can be directed to the corresponding author.

## References

[1] Pereira-Pinto R., Moreira O., Vaz-Velho M. (2025). Managing boar taint: Issues, prevention strategies, and detection methods. Anim. Sci. J..

[2] Wesoly R., Weiler U. (2012). Nutritional influences on skatole formation and skatole metabolism in the pig. Animals.

[3] Annor-Frempong I.E., Nute G.R., Whittington F.W., Wood J.D. (1997). The problem of taint in pork: 1. Detection thresholds and odour profiles of androstenone and skatole in a model system. Meat Sci..

[4] Font-i-Furnols M., Guerrero L., Serra X., Rius M.A.À., Oliver M.A.À. (2000). Sensory characterization of boar taint in entire male pigs. J. Sens. Stud..

[5] Weiler U., Font-i-Furnols M., Fischer K., Kemmer H., Oliver M.A., Gispert M., Dobrowolski A., Claus R. (2000). Influence of differences in sensitivity of Spanish and German consumers to perceive androstenone on the acceptance of boar meat differing in skatole and androstenone concentrations. Meat Sci..

[6] Klyuchnikova M.A., Kvasha I.G., Laktionova T.K., Voznessenskaya V.V. (2022). Olfactory perception of 5α-androst-16-en-3-one: Data obtained in the residents of central Russia. Data Brief.

[7] Wysocki C.J., Dorries K.M., Beauchamp G.K. (1989). Ability to perceive androstenone can be acquired by ostensibly anosmic people. Proc. Natl. Acad. Sci. USA.

[8] Bonneau M., Chevillon P. (2012). Acceptability of entire male pork with various levels of androstenone and skatole by consumers according to their sensitivity to androstenone. Meat Sci..

[9] Garrido M.D., Egea M., Linares M.B., Martínez B., Viera C., Rubio B., Borrisser-Pairó F. (2016). A procedure for sensory detection of androstenone in meat and meat products from entire male pigs: Development of a panel training. Meat Sci..

[10] Prusa K., Nederveld H., Runnels P.L., Li R., King V.L., Crane J.P. (2011). Prevalence and relationships of sensory taint, 5α-androstenone and skatole in fat and lean tissue from the loin (Longissimus dorsi) of barrows, gilts, sows, and boars from selected abattoirs in the United States. Meat Sci..

[11] Pereira-Pinto R., Barbosa C., Mata F., Reis N., Barros D., Vaz-Velho M. (2024). Assessing boar taint in Portuguese pork: A small-scale study of prevalence and classification via established detection thresholds. Span. J. Agric. Res..

[12] Squires E.J., Bone C., Cameron J. (2020). Pork production with entire males: Directions for control of boar taint. Animals.

[13] Bonneau M., Weiler U. (2019). Pros and cons of alternatives to piglet castration: Welfare, boar taint, and other meat quality traits. Animals.

[14] van Ferneij J.P. The Pig Castration Situation in the European Union. https://www.3tres3.com/en/articles/the-pig-castration-situation-in-the-european-union_18100/.

[15] Zamaratskaia G., Squires E.J. (2009). Biochemical, nutritional and genetic effects on boar taint in entire male pigs. Animal.

[16] Zhang M., Liao X., Wang F., Shen H., Mao S., Xu Z. (2026). Decoding skatole: A comprehensive review on biosynthesis, metabolism, and mitigation in livestock production. Bioresour. Technol..

[17] Duarte D.A.S., Schroyen M., Mota R.R., Vanderick S., Gengler N. (2021). Recent genetic advances on boar taint reduction as an alternative to castration: A review. J. Appl. Genet..

[18] Tholen E. Breeding for Reduced Boar Taint. https://lohmann-breeders.com/lohmanninfo/breeding-for-reduced-boar-taint/.

[19] Bilić-Šobot D., Čandek-Potokar M., Kubale V., Škorjanc D. (2014). Boar taint: Interfering factors and possible ways to reduce it. Agricultura.

[20] Xue J., Dial G.D., Holton E.E., Vickers Z., Squires E.J., Lou Y., Godbout D., Morel N. (1996). Breed differences in boar taint: Relationship between Tissue levels boar taint compounds and sensory analysis of taint. J. Anim. Sci..

[21] Ciobanu D.C., Lonergan S.M., Huff-Lonergan E.J. (2011). Genetics of Meat Quality and Carcass Traits.

[22] Botelho M.E., Lopes M.S., Mathur P.K., Knol E.F., e Silva F.F., Lopes P.S., Gimarães S.E.F., Marques D.B.D., Veroneze R. (2022). Weighted genome-wide association study reveals new candidate genes related to boar taint compounds 1. Livest. Sci..

[23] Kušec D., Cimerman E., Škrlep M., Karolyi D., Gvozdanović K., Komlenić M., Radišić Ž., Kušec G. (2021). Influence of immunocastration on slaughter traits and boar taint compounds in pigs originating from three different terminal sire lines. Animals.

[24] Prunier A., Brillouët A., Merlot E., Meunier-Salaün M.C., Tallet C. (2013). Influence of housing and season on pubertal development, boar taint compounds and skin lesions of male pigs. Animal.

[25] Urbanová D., Stupka R., Okrouhlá M., Čítek J., Vehovsky K., Zadinová K. (2016). Nutritional effects on boar taint in entire male pigs: A review. Sci. Agric. Bohem..

[26] Hansen L.L., Larsen A.E., Jensen B.B., Hansen-Møller J. (1997). Short time effect of zinc bacitracin and heavy fouling with faeces plus urine on boar taint. Anim. Sci..

[27] Giersing M., Ladewig J., Forkman B. (2006). Animal welfare aspects of preventing boar taint. Acta Vet. Scand..

[28] Byrne D.V., Thamsborg S.M., Hansen L.L. (2008). A sensory description of boar taint and the effects of crude and dried chicory roots (*Cichorium intybus* L.) and inulin feeding in male and female pork. Meat Sci..

[29] Li X., Jensen Bent B., Canibe N. (2019). The mode of action of chicory roots on skatole production in entire male pigs is neither via reducing the population of skatole-producing bacteria nor via increased butyrate production in the hindgut. Appl. Environ. Microbiol..

[30] Hawe S.M., Walker N., Moss B.W. (1992). The effects of dietary fibre, lactose and antibiotic on the levels of skatole and indole in faeces and subcutaneous fat in growing pigs. Anim. Sci..

[31] Øverland M., Kjos N.K., Fauske A.K., Teige J., Sørum H. (2011). Easily fermentable carbohydrates reduce skatole formation in the distal intestine of entire male pigs. Livest. Sci..

[32] Hansen L.L., Larsen A.E., Jensen B.B., Hansen-Møller J., Barton-Gade P. (1994). Influence of stocking rate and faeces deposition in the pen at different temperatures on skatole concentration (boar taint) in subcutaneous fat. Anim. Sci..

[33] Heyrman E., Millet S., Tuyttens F.A.M., Ampe B., Janssens S., Buys N., Wauters J., Vanhaecke L., Aluwé M. (2017). Olfactory evaluation of boar taint: Effect of factors measured at slaughter and link with boar taint compounds. Animal.

[34] Schally A.V., Arimura A., Baba Y., Nair R.M.G., Matsuo H., Redding T.W., Debeljuk L., White W.F. (1971). Isolation and properties of the FSH and LH-releasing hormone. Biochem. Biophys. Res. Commun..

[35] Campal-Espinosa A.C., Junco-Barranco J.A., Fuentes-Aguilar F., Calzada-Aguilera L., Rivacoba-Betancourt A., Rodríguez-Bueno R.H., Bover-Campal A.C., Bover-Fuentes E.E., González L., de Quesada L. (2023). Influence of humoral response against GnRH, generated by immunization with a therapeutic vaccine candidate on the evolution of patients with castration-sensitive prostate adenocarcinoma. Technol. Cancer Res. Treat..

[36] Kress K.B. (2020). Immunocastration of Male Pigs.

[37] Schwarzenberger F., Krawinkel P., Jeserschek S.M., Schauerte N., Geiger C., Balfanz F., Knauf-Witzens T., Sicks F., Martinez Nevado E., Anfray G. (2022). Immunocontraception of male and female giraffes using the GnRH vaccine Improvac^®^. Zoo Biol..

[38] Kirkpatrick J.F., Lyda R.O., Frank K.M. (2011). Contraceptive vaccines for wildlife: A review. Am. J. Reprod. Immunol..

[39] Kawase K., Tomiyasu J., Ban K., Ono R., Ando S., Ono A., Kimura R., Tomisawa K., Matsui M., Shiihara S.I. (2021). Contraceptive effect of a gonadotropin-releasing hormone vaccine on a captive female African Lion (*Panthera leo*): A case study. J. Vet. Med. Sci..

[40] D’Souza D.N., Hewitt R.J.E., van Barneveld R.J. (2018). Pork production with entire males and immunocastrates in Australia. Adv. Anim. Biosci..

[41] Čandek-Potokar M., Škrlep M., Zamaratskaia G., Payan-Carreira R. (2017). Immunocastration as alternative to surgical castration in pigs. Theriogenology.

[42] Mancini M., Menozzi D., Arfini F. (2017). Immunocastration: Economic implications for the pork supply chain and consumer perception. An assessment of existing research. Livest. Sci..

[43] Backus G., Higuera M., Juul N., Nalon E., de Briyne N. (2018). Second Progress Report 2015–2017 on the European Declaration on Alternatives to Surgical Castration of Pigs.

[44] Agency E.M. Improvac: EPAR—Product information. https://www.ema.europa.eu/en/medicines/veterinary/EPAR/improvac.

[45] U.S. Environmental Protection Agency GonaCon Immunocontraceptive Vaccine (EPA Reg. No. 56228-40).

[46] Škrlep M., Tomašević I., Mörlein D., Novaković S., Egea M., Garrido M.D., Linares M.B., Peñaranda I., Aluwé M., Font-i-Furnols M. (2020). The use of pork from entire male and immunocastrated pigs for meat products-an overview with recommendations. Animal.

[47] Miller L.A., Fagerstone K.A. (2000). Induced infertility as a wildlife management tool. Proc. Vertebr. Pest Conf..

[48] Miller L.A., Rhyan J., Killian G. GonaCon™, a versatile GnRH contraceptive for a large variety of pest animal problems. Proceedings of the 21st Vertebrate Pest Conference.

[49] Miller L.A., Gionfriddo J.P., Fagerstone K.A., Rhyan J.C., Killian G.J. (2008). The single-shot gnrh immunocontraceptive vaccine (gonacon™) in white-tailed deer: Comparison of several gnrh preparations. Am. J. Reprod. Immunol..

[50] Davis H.L., Weeratna R., Dominowski P.J. (2013). Vaccines Comprising Cholesterol and CpG as Sole Adjuvant-Carrier Molecules.

[51] Monleón E., Noya A., Carmen Garza M., Ripoll G., Sanz A. (2020). Effects of an anti-gonadotrophin releasing hormone vaccine on the morphology, structure and function of bull testes. Theriogenology.

[52] Botelho-Fontela S., Paixão G., Pereira-Pinto R., Vaz-Velho M., Pires M.A., Payan-Carreira R., Patarata L., Lorenzo J.M., Silva A., Esteves A. (2024). The effects of different immunocastration protocols on meat quality traits and boar taint compounds in male Bísaro pigs. Theriogenology.

[53] Ahmed S., Jiang X., Liu G., Sadiq A., Farooq U., Wassie T., Saleem A.H., Zubair M. (2022). New trends in immunocastration and its potential to improve animal welfare: A mini review. Trop. Anim. Health Prod..

[54] Zamaratskaia G., Rydhmer L., Andersson H.K., Chen G., Lowagie S., Andersson K., Lundström K. (2008). Long-term effect of vaccination against gonadotropin-releasing hormone, using Improvac™, on hormonal profile and behaviour of male pigs. Anim. Reprod. Sci..

[55] Scheid I.R., Oliveira F.T.T., Borges A.C., Braga T.F., Soncini R.A., Mathur S., Allison J.R., Hennessy D.P. (2014). A single dose of a commercial anti-gonadotropin releasing factor vaccine has no effect on testicular development, libido, or sperm characteristics in young boars. J. Swine Health Prod..

[56] Dunshea F.R., Colantoni C., Howard K., McCauley I., Jackson P., Long K.A., Lopaticki S., Nugent E.A., Simons J.A., Walker J. (2001). Vaccination of boars with a GnRH vaccine (Improvac) eliminates boar taint and increases growth performance. J. Anim. Sci..

[57] Livingstone P.G., Hancox N., Nugent G., de Lisle G.W. (2015). Toward eradication: The effect of *Mycobacterium bovis* infection in wildlife on the evolution and future direction of bovine tuberculosis management in New Zealand. N. Z. Vet. J..

[58] Harris E.K., Mellencamp M.A., Johnston L.J., Cox R.B., Shurson G.C. (2017). Effect of time interval between the second Improvest^®^ dose and slaughter and corn dried distillers grains with solubles feeding strategies on carcass composition, primal cutout, and pork quality of immunologically castrated pigs. Meat Sci..

[59] Lealiifano A.P., Pluske J., Dunshea F., Mullan B. (2009). Altering the Timing of an Immunocastration Vaccine (Improvac^®^) to Reduce Its Impact on Attributes of Pig Performance.

[60] Pearce M., Andrews S., Brock F.C., Allison J.R.D. Effects of vaccination with Improvac^®^ on boar taint and carcase quality of male pigs reared under commercial conditions in Europe. Proceedings of the 55th International Congress of Meat Science and Technology.

[61] Control W.F. GonaCon™. https://wildlifefertilitycontrol.org/wp-content/uploads/2025/03/GONACON.pdf.

[62] Benka V.A., Levy J.K. (2015). Vaccines for feline contraception: GonaCon GnRH-hemocyanin conjugate immunocontraceptive. J. Feline Med. Surg..

[63] United States Department of Agriculture, Animal and Plant Health Inspection Service, Wildlife Services (2022). The Use of GonaCon in Wildlife Damage Management; Chapter XI. Human Health and Ecological Risk Assessment for the Use of Wildlife Damage Management Methods.

[64] Wicks N., Crouch S., Pearl C.A. (2013). Effects of Improvac and Bopriva on the testicular function of boars ten weeks after immunization. Anim. Reprod. Sci..

[65] Janett F., Stump R., Burger D., Thun R. (2009). Suppression of testicular function and sexual behavior by vaccination against GnRH (Equity™) in the adult stallion. Anim. Reprod. Sci..

[66] Elhay M., Newbold A., Britton A., Turley P., Dowsett K., Walker J. (2007). Suppression of behavioural and physiological oestrus in the mare by vaccination against GnRH. Aust. Vet. J..

[67] Aurich C., Kaps M. (2022). Suppression of reproductive behaviour and gonadal function in female horses—An update. Reprod. Domest. Anim..

[68] Janett F., Gerig T., Tschuor A.C., Amatayakul-Chantler S., Walker J., Howard R., Bollwein H., Thun R. (2012). Vaccination against gonadotropin-releasing factor (GnRF) with Bopriva significantly decreases testicular development, serum testosterone levels and physical activity in pubertal bulls. Theriogenology.

[69] Rocha L.F., Souza R.S., Santana A.L.A., Macedo D.S., Santana A.M.S., da Silva R.C., Bezerra P.A., de Jesus R.D.L., Barbosa L.P. (2021). Reproductive parameters of lambs immunocastrated with anti-GnRH vaccine. Anim. Reprod..

[70] Brunius C., Zamaratskaia G., Andersson K., Chen G., Norrby M., Madej A., Lundström K. (2011). Early immunocastration of male pigs with Improvac^®^—Effect on boar taint, hormones and reproductive organs. Vaccine.

[71] Werner D., Baldinger L., Bussemas R., Büttner S., Weißmann F., Ciulu M., Mörlein J., Mörlein D. (2021). Early immunocastration of pigs: From farming to meat quality. Animal.

[72] Zeng F., Ding Y., Wassie T., Jing H., Ahmed S., Liu G., Jiang X. (2022). Recent advances in immunocastration in sheep and goat and its animal welfare benefits: A review. J. Integr. Agric..

[73] (2019). European Cooperation in Science and Technology Action CA15215, Innovative Approaches in Pork Production with Entire Males Core Group. Fact Sheet: Pork Production with Entire Males. https://www.ca-ipema.eu/download/415/documents/publications/Factsheet_Boars_8-2019.pdf.

[74] De Briyne N., Berg C., Blaha T., Temple D. (2016). Pig castration: Will the EU manage to ban pig castration by 2018?. Porc. Health Manag..

[75] Pfizer Animal Health Improvac—The Mexican Experience. https://www.improvac.com/.

[76] Pesenti Rossi G., Dalla Costa E., Filipe J.F.S., Mazzola S.M., Motta A., Borciani M., Gastaldo A., Canali E., Pilia F., Argenton M. (2022). Does immunocastration affect behaviour and body lesions in heavy pigs?. Vet. Sci..

[77] Animal Health Australia (2016). Australian Animal Welfare Standards and Guidelines for Pigs.

[78] National Farm Animal Care Council (2014). Code of Practice for the Care and Handling of Pigs.

[79] National Animal Welfare Advisory Committee (2018). Code of Welfare: Pigs.

[80] United States Food and Drug Administration (2011). Freedom of Information Summary: Original New Animal Drug Application NADA 141-322, Improvest; NADA 141-322.

[81] Wang L., Li D. (2024). Current status, challenges and prospects for pig production in Asia. Anim. Biosci..

[82] Čandek-Potokar M., Batorek Lukač N. (2015). Alternatives to surgical castration in pigs. Bulg. J. Anim. Husb..

[83] Needham T., Lambrechts H., Hoffman L.C. (2017). Castration of male livestock and the potential of immunocastration to improve animal welfare and production traits: Invited Review. S. Afr. J. Anim. Sci..

[84] Atallah E., Pesenti Rossi G., Soares Filipe J.F., Dalla Costa E., Mazzola S.M., Minero M., Pecile A., Motta A., Barbieri S. (2025). Assessing salivary cortisol and testosterone as non-invasive biomarkers for GnRH-immunocastration efficiency in heavy pigs. BMC Vet. Res..

[85] Kress K., Millet S., Labussière É., Weiler U., Stefanski V. (2019). Sustainability of pork production with immunocastration in europe. Sustainability.

[86] Staff Zoetis Receives European Commission Marketing Authorization for IMPROVAC^®^ in Female Pigs. https://www.3tres3.com/en/company_news/zoetis-receives-ec-marketing-authorization-for-improvac%C2%AE-in-females_18930/.

[87] Baumgartner J., Laister S., Koller M., Pfützner A., Grodzycki M., Andrews S., Schmoll F. (2010). The behaviour of male fattening pigs following either surgical castration or vaccination with a GnRF vaccine. Appl. Anim. Behav. Sci..

[88] Vanhonacker F., Verbeke W., Tuyttens F.A.M. (2009). Belgian consumers’ attitude towards surgical castration and immunocastration of piglets. Anim. Welf..

[89] Vanhonacker F., Verbeke W. (2011). Consumer response to the possible use of a vaccine method to control boar taint v. physical piglet castration with anaesthesia: A quantitative study in four European countries. Animal.

[90] Di Pasquale J., Vecchio Y., Martelli G., Sardi L., Adinolfi F., Nannoni E. (2020). Health risk perception, consumption intention, and willingness to pay for pig products obtained by immunocastration. Animal.

[91] Aluwé M., Heyrman E., Kostyra E., Żakowska-Biemans S., Almeida J., Citek J., Font-i-Furnols M., Moreira O., Zadinová K., Tudoreanu L. (2022). Consumer evaluation of meat quality from barrows, immunocastrates and boars in six countries. Animal.

[92] Font-i-Furnols M., Claret A., Guerrero L., Dalmau A. (2022). Consumers’ expectations about meat from surgical castrated or immunocastrated male and female iberian pigs. Animals.

[93] Di Pasquale J., Nannoni E., Sardi L., Rubini G., Salvatore R., Bartoli L., Adinolfi F., Martelli G. (2019). Towards the abandonment of surgical castration in pigs: How is immunocastration perceived by italian consumers?. Animals.

[94] Australian Pork Limited (2024). Evaluation of the Timing and Dosage of Improvac^®^ Vaccine and Effectiveness in Pigs of Differing Liveweights to Control Boar Taint Compounds and Pork Quality.

[95] Aida V., Pliasas V.C., Neasham P.J., North J.F., McWhorter K.L., Glover S.R., Kyriakis C.S. (2021). Novel vaccine technologies in veterinary medicine: A herald to human medicine vaccines. Front. Vet. Sci..

[96] Shalash A.O., Wang W., Xia Y., Hussein W.M., Bashiri S., D’Occhio M.J., Stephenson R.J., Skwarczynski M., Toth I. (2025). Evaluation of novel single-dose vaccine candidates against gonadotropin-releasing hormone (GnRH) in mice. Vaccine.

[97] Faruck M.O., Koirala P., Yang J., D’Occhio M.J., Skwarczynski M., Toth I. (2021). Polyacrylate-GnRH peptide conjugate as an oral contraceptive vaccine candidate. Pharmaceutics.

[98] Trimzi M.A., Ham Y.B. (2021). A needle-free jet injection system for controlled release and repeated biopharmaceutical delivery. Pharmaceutics.

[99] Ikechukwu P., Agu R. (2026). Advancing needle-free jet injectors for global vaccine delivery. Pharmaceutics.

[100] Sonoda J., Mizoguchi I., Inoue S., Watanabe A., Sekine A., Yamagishi M., Miyakawa S., Yamaguchi N., Horio E., Katahira Y. (2023). A promising needle-free pyro-drive jet injector for augmentation of immunity by intradermal injection as a physical adjuvant. Int. J. Mol. Sci..

[101] Wu Y., Ren B., Gao X., Li N., Lin L., Li Q. (2025). Evaluation of the immunization effects of needle-free injection for four common porcine vaccines. Vaccine.

[102] Heyrman E., Kowalski E., Millet S., Tuyttens F.A.M., Ampe B., Janssens S., Buys N., Wauters J., Vanhaecke L., Aluwé M. (2019). Monitoring of behavior, sex hormones and boar taint compounds during the vaccination program for immunocastration in three sire lines. Res. Vet. Sci..

[103] Kim Y.C., Jarrahian C., Zehrung D., Mitragotri S., Prausnitz M.R. (2012). Delivery systems for intradermal vaccination. Curr. Top. Microbiol. Immunol..

[104] Temple D., Jiménez M., Escribano D., Martín-Valls G., Díaz I., Manteca X. (2020). Welfare benefits of intradermal vaccination of piglets. Animal.

[105] Lycke N. (2012). Recent progress in mucosal vaccine development: Potential and limitations. Nat. Rev. Immunol..

[106] Ji M., Nie J., Chen Y., Gang J., Dong C., Huang H., Liu L.-M., Zhou Y. (2026). Advances and future applications of immunocastration techniques: A comprehensive review. npj Vaccines.

[107] Liu M.A. (2019). A comparison of plasmid DNA and mRNA as vaccine technologies. Vaccines.

[108] Verbeke R., Lentacker I., De Smedt S.C., Dewitte H. (2019). Three decades of messenger RNA vaccine development. Nano Today.

[109] Kutzler M.A., Weiner D.B. (2008). DNA vaccines: Ready for prime time?. Nat. Rev. Genet..

[110] Skwarczynski M., Toth I. (2016). Peptide-based synthetic vaccines. Chem. Sci..

[111] Heegaard P.M.H., Dedieu L., Johnson N., Le Potier M.-F., Mockey M., Mutinelli F., Vahlenkamp T., Vascellari M., Sørensen N.S. (2011). Adjuvants and delivery systems in veterinary vaccinology: Current state and future developments. Arch. Virol..

[112] Foged C., Hansen J., Agger E.M. (2012). License to kill: Formulation requirements for optimal priming of CD8(+) CTL responses with particulate vaccine delivery systems. Eur. J. Pharm. Sci..

